# Neurosarcoidosis: a review from diagnosis and future perspectives

**DOI:** 10.1016/j.eclinm.2025.103653

**Published:** 2025-11-17

**Authors:** Willeke F. Westendorp, Diederik van de Beek, Matthijs C. Brouwer

**Affiliations:** Amsterdam UMC, University of Amsterdam, Department of Neurology, Amsterdam Neuroscience, Amsterdam, the Netherlands

**Keywords:** Sarcoidosis, Myelitis, Meningitis, Cranial nerve palsy, Immunosuppressive treatment, TNF-α antagonists

## Abstract

Sarcoidosis is complicated by neurosarcoidosis in 5–15% of cases. Neurosarcoidosis is characterized by the presence of non-necrotizing granulomas that can affect any part of the nervous system. Key clinical presentations include chronic meningitis, cranial nerve palsies, parenchymal cerebral and spinal lesions, peripheral neuropathy, or myopathy. Diagnosis requires histopathological confirmation of granulomatous disease and exclusion of other potential diagnoses. Immunosuppressive treatment strategies are based on cohort studies and expert consensus, as randomized controlled trials are lacking. Neurosarcoidosis is associated with high morbidity, with only one third of patients being symptom-free status after treatment. Optimal care should be centralized to provide specialized multidisciplinary expertise and facilitate research. Future research should focus on the optimal timing of 3rd line treatment initiation and treatment personalization.

**Funding:**

The Netherlands Organisation for Health Research and Development (ZonMw), 10.13039/501100000781European Research Council.

**Search strategy and selection criteria**

Fig. 1 shows the search strategy. We identified relevant articles by searching Pubmed between 1967 and 2025 and screening references from relevant articles, the final reference list was generated on the basis of the relevance to the topics in this review.

## Introduction

Sarcoidosis is characterized by the presence of non-necrotizing granulomas that can affect any part of the human body, including the nervous system. Neurosarcoidosis encompasses a range of manifestations, such as cranial nerve palsies, chronic meningitis, central nervous system inflammatory lesions, neuropathy, and myopathy (Fig. 2). Diagnosis of neurosarcoidosis is often challenging as the clinical presentation of the disease and results of ancillary investigations can be non-specific. The condition causes substantial morbidity, with only one-third of achieving a symptom-free status after treatment.^1^ In this review, we summarize current knowledge on neurosarcoidosis, discuss the evidence for the different treatment options, and highlight goals for future research.

**Fig. 1:**
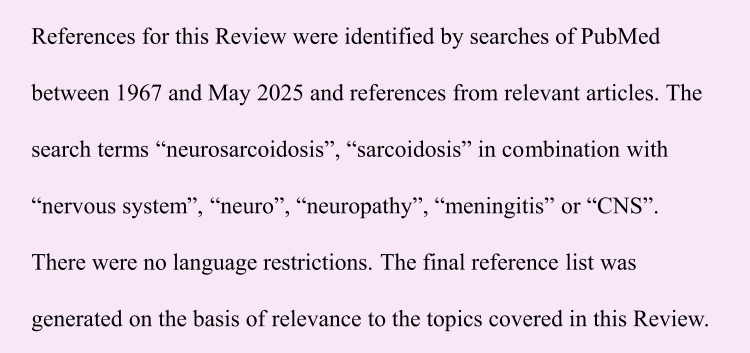
Search strategy and selection criteria.

**Fig. 2:**
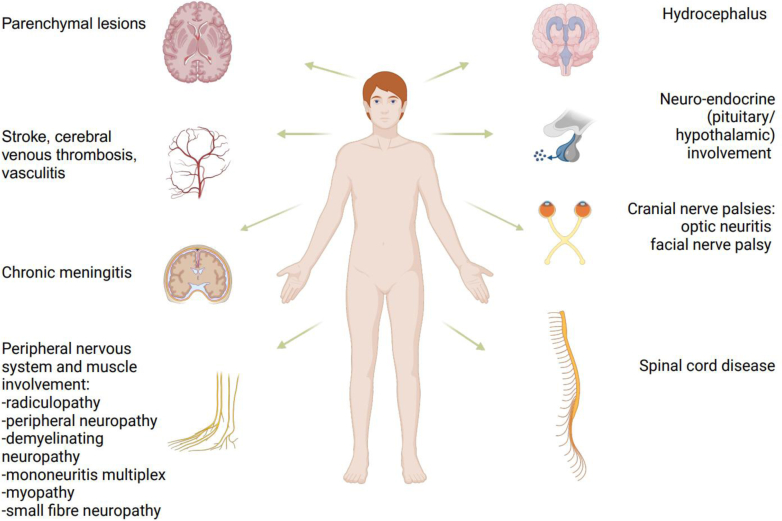
Manifestations of neurosarcoidosis.

## Epidemiology

Sarcoidosis affects 1–40/100,000 patients per year and incidence is highest in Northern Europe (11–24/100,000) and among African Americans (18–71/100,000).^2^ Neurosarcoidosis is estimated to occur in 5–15% of sarcoidosis patients, and in 50–70% of neurosarcoidosis patients, neurologic disease is the first manifestation of sarcoidosis. Presentation with isolated neurosarcoidosis occurred in 22% of patients.^1^

## Pathophysiology

Sarcoidosis is thought to originate from an exaggerated immune response to an unknown antigen in patients with genetic susceptibility, combined with exposition to certain environmental factors. Heritability of sarcoidosis was estimated at 39–66% in two Scandinavian studies.^3^^,^^4^ Sarcoidosis risk is increased 3.7 fold with one affected first-degree relative, 4.7 fold with two affected relatives and 80-fold in co-twins of affected monozygotic siblings.^3^^,^^4^ Many genetic studies have detected gene variants involved in the immune system associated with sarcoidosis risk.^5^ The strongest genetic associations for increased risk or greater severity of sarcoidosis is with certain human leukocyte antigen (HLA) subtypes.

For African Americans with sarcoidosis, first-degree relatives have about a 3-fold higher risk of the disease.^6^ Different studies on the genetic susceptibility in these patients shows that disease risk and presentation are likely influenced by more than one gene.^6^^,^^7^

For neurosarcoidosis, associations with HLA subtypes were found in a Japanese population, but not in UK or Dutch populations. Also, a genetic variant in the zinc finger protein 592 gene was detected, possibly involved in T-cell immune response, that was associated with neurosarcoidosis in African-Americans.^8^ In an article on an index patient with treatment-resistant neurosarcoidosis within a family with a sarcoidosis rate of 20%, 34 genes were identified with a possible role in development of sarcoidosis.^9^ Three genes - JAK2, BACH2, and NCF1–were identified with known associations with inflammatory bowel disease and chronic granulomatous disease.^9^ The finding that the genetic variants associated with neurosarcoidosis are related to the antigen-presenting processes and to specific T-helper inflammatory reactions, is in line with the theories on immunological pathophysiology. The contribution of each of these genes to the development of neurosarcoidosis needs further study, but it can be concluded this risk is polygenetic.

Other factors, such as environmental and infectious agents, are thought to be crucial for the development of sarcoidosis. The frequent involvement of lungs, eyes, and skins is considered a key indicator for environmental exposure as a potential cause. Numerous environmental factors have been identified, including occupational exposure, seasonal related peaks of sarcoidosis incidence and personal factors (patients are less likely to be smokers).^10^ Infectious agents may also play a role, with some evidence suggesting involvement of *Mycobacterium* species and ***Cutibacterium acnes***, though their exact contribution to the disease's pathogenesis remains unclear.^11^

## Immunology

Although not specific for the disease, one of the main immunological and pathological hallmarks of neurosarcoidosis is the formation of non-necrotizing granuloma. Macrophages, seen early in interstitial lung disease, and dendritic cells play an important role in the development of granulomas.^2^^,^^12^ In lung biopsy samples of sarcoid granulomas, the central part of granulomas consisted of epithelioid cells with surrounding giant cells, lymphocytes, macrophages, and plasma cells.^13^ Epithelioid cells are large polygonal cells derived from macrophages, but without phagocytic activity, that secrete ACE and TNF-α.^14^, ^15^, ^16^ TNF-α is a cytokine deemed crucial for granuloma formation and therefore a main target for therapy.^17^ From an evolutionary point of view, granulomas might be aimed at isolating a persistent endogenous or exogenous antigen thereby causing chronic inflammation.^2^

Apart from non-necrotizing granulomas, another immunological hallmark of sarcoidosis is a polarized inflammatory response towards CD4-positive T helper cells. Macrophages and dendritic cells secrete cytokines inducing CD4 Th cells to differentiate into Th1 and Th17.1 effector cells.^2^ Evidence for T-cell involvement comes from analysis of lymphocyte populations in BAL-fluid and affected tissues in sarcoidosis patients, from involvement of the STAT3 signalling pathway (important in differentiation of Th17 cells) and from the observation that HIV infection and subsequent CD4 lymphopenia leads to remission of sarcoidosis.^2^ Analogue to the findings in BAL-fluid, three studies (with 11, 48, and 66 neurosarcoidosis patients) found an elevated (≥five) or increased CD4:CD8 ratio in CSF, and one study found a normal ratio in five of eight evaluated neurosarcoidosis patients.^18^, ^19^, ^20^, ^21^ Single-cell RNA sequencing of CSF and blood in six probable neurosarcoidosis (4 treatment naïve) patients found interferon-mediated clonal CD8 T cell expansion instead of CD4 T cell expansion, but findings need to be confirmed.^22^

### Clinical presentation

Neurosarcoidosis can affect any part of the nervous system and cause a wide range of symptoms. In a meta-analysis of 29 articles describing 1088 possible, probable, or definite neurosarcoidosis patients, most common symptoms were headache (32%), sensory abnormalities (29%), and limb paresis (19%). The most common clinical manifestations were cranial nerve palsies, chronic meningitis, cerebral parenchymal lesions, spinal cord disease, and peripheral nervous system involvement (Fig. 2).^1^ Although in approximately half of patients neurological symptoms were the first manifestation of sarcoidosis, 84% of patients experienced manifestation(s) outside the nervous system at some point during disease course. Most frequently the lungs (57%), eyes (20%), lymph nodes (18%), skin, and joints (both 18%) were affected.

## Cranial nerve palsies

Cranial neuropathies occurred in 55% of neurosarcoidosis patients; the optic and facial nerve are most often involved (21 and 24% respectively) but involvement of other (trigeminal nerve in 12%) or multiple (28%) cranial nerves is not uncommon.^1^ Optic neuropathy can be caused by compression from a granulomatous lesion, by increased intracranial pressure or by optic neuritis. Patients with optic neuritis caused by sarcoidosis less often experience pain as compared to a classical optic neuritis (20–30% vs. > 60%), more often have bilateral involvement and an abnormal ophthalmologic evaluation.^23^ Prognosis is poor as only 40% of patients improve after corticosteroid therapy.^23^

Facial nerve palsies are common as a first presentation of neurosarcoidosis and occur in isolation of other neurologic involvement in approximately half of patients.^24^ The onset is usually acute, the majority unilateral, and, in one third of cases, other cranial nerves are affected as well.^24^ Facial nerve palsy in sarcoidosis patients can also occur as part of the Heerfordt syndrome, in which it occurs in conjunction with parotid swelling, uveitis, and fever. Sarcoidosis is one of the main causes of bilateral facial nerve palsy, but other causes, such neuroborreliosis, Guillain-Barré-syndrome, and Melkersson-Rosenthal syndrome need to be considered. Outcome of facial nerve function in neurosarcoidosis is favorable in most cases.^24^

## Meningitis and hydrocephalus

Neurosarcoidosis can affect the pachy- or leptomeninges and cause a subacute or chronic meningitis. Chronic meningitis is defined as signs and symptoms caused by inflammation of the meninges which persist for at least 4 weeks without spontaneous resolution.^25^ Typical complaints are headache, lethargy, mental status changes, and fever.^25^ In sarcoidosis-related chronic meningitis, cranial MRI scanning often shows leptomeningeal enhancement or pachymeningeal involvement with diffuse dural thickening and focal dural masses with irregular/nodular aspect.^26^ Chronic meningitis can be complicated by cranial nerve palsies and hydrocephalus or, less commonly, intracranial hypertension. Hydrocephalus mostly presents with headache, is obstructive in 90% of cases and in 80–90% neurosurgical intervention is needed.^27^ Therefore, treatment monitoring must include comparison of ventricle size with previous exams.

## Spinal cord disease

Myelitis in neurosarcoidosis most often presents with sensory disturbances (85%), loss of strength (76%) and micturition abnormalities (73%).^28^ Onset can be insidious and diagnosis is often delayed: the median time from symptom onset to neurologist consultation was 13 weeks and median time to diagnosis was seven months in 41 patients with sarcoidosis-associated myelitis.^28^ MRI typically shows a longitudinally extensive myelitis (≥ three segments) with contrast enhancement on MRI, but can also show leptomeningeal spread and reactive edema in the spinal cord, or the Köbner phenomenon (lesion in front of discopathies with false appearance of compressive myelopathy).^29^ CSF pleocytosis is present in the majority of patients. The cauda equina can be involved as well, often with nodular enhancement on MRI. A third of myelitis patients experience relapses and need prolonged immunosuppressive treatment. Many patients have permanent disability: in 41 patients with sarcoidosis-associated myelitis 40% needed a walking aid and 20% were unable to walk.^28^

## Cerebral parenchymal lesions

**Fig. 3:**
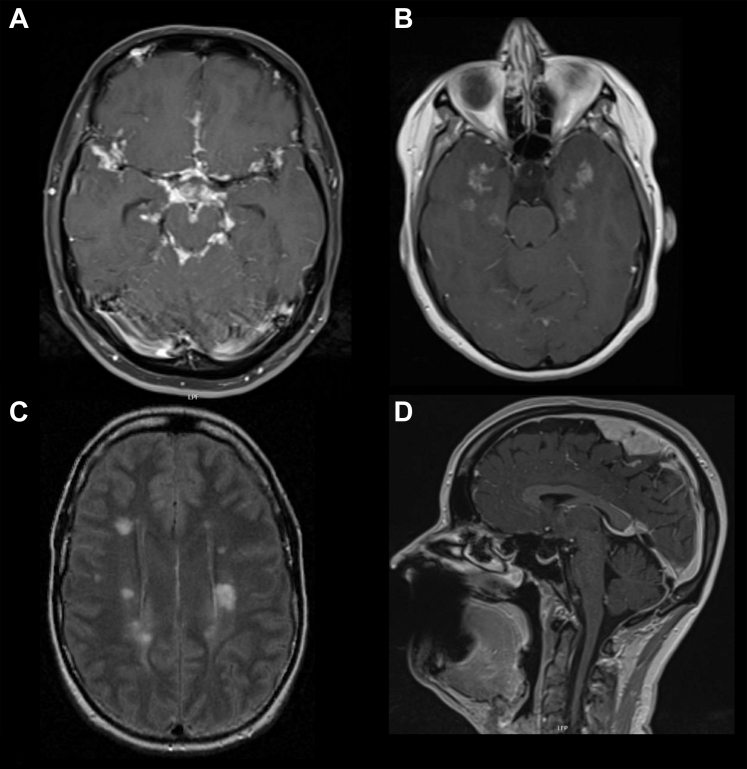
Parenchymal and meningeal involvement in neurosarcoidosis. A: 32 yo female with fatigue and headache, biopsy confirmed sarcoidosis MRI T1 + Gd showing leptomeningeal enhancement. B: 28 yo female with biopsy confirmed sarcoidosis and migraine, cranial MRI, T1 + Gd showing parenchymal enhancement in temporal lobe. C: 40 yo female with initial diagnosis of multiple sclerosis, MRI FLAIR showing white matter lesions, later diagnosed as neurosarcoidosis based on biopsy confirmed sarcoidosis. D: 37 yo female with focal epilepsy and extra-axial lesion suspected to be meningeoma or metastasis, MRI T1 + Gd showing homogeneous enhancing mass adjacent to the falx, which proved to be definite neurosarcoidosis after resection.

Cerebral parenchymal involvement can be subdivided in extension of leptomeningeal involvement into perivascular spaces, intracerebral lesions, white matter lesions, and intracerebral noduli (Fig. 3). Patients with tumefactive cerebral parenchymal lesions typically present with headache, cognitive deficit, or seizures. Cranial MRI often shows a stellate or spherical morphology of the lesion(s) on MRI with concomitant leptomeningeal enhancement.^30^

## Peripheral nervous system (PNS) disease and muscular sarcoidosis

In a meta-analysis PNS disease was present in 17% of neurosarcoidosis patients (97 of 558) and consisted of polyneuropathy in 11% (56 of 487), multiple mononeuropathies in 12% (16 of 137), and radiculopathy in 5% (7 of 140).^1^ In 11 cases with peripheral nerve involvement treatment resulted in complete recovery in five, partial improvement in one and no response in five patients.^31^

Myopathy in sarcoidosis is rare and mostly manifests as subacute or chronic muscle weakness or myalgia.^32^ A ‘tiger man’ appearance on MRI or PET-CT scan, due to focal intramuscular masses, is typical for neurosarcoidosis.^32^ Despite treatment about half of 12 patients with muscular involvement of sarcoidosis had substantial morbidity at follow-up.

Small fibre neuropathy (SFN) is a common complication of sarcoidosis and associated with non-length dependent pain, sensory disturbances, and dysautonomia.^33^ SFN is seen in many other auto-immune diseases but exact pathogenesis is unknown.

## Vascular involvement

Small vessel ischaemic stroke is sometimes seen in neurosarcoidosis, often concomitant leptomeningitis or cranial nerve involvement is present.^34^ Micro- or macro hemorrhages are occasionally seen. Causes of stroke in neurosarcoidosis include vasculitis, venous phlebitis, or emboli from (sarcoid) cardiac disease or traditional atherosclerosis. The typical pattern of perivascular radial enhancement, as seen in GFAP astrocytopathy, cerebral vasculitis, and intravascular lymphoma, can occur in neurosarcoidosis as well.^35^ Vascular involvement in neurosarcoidosis might be more common than suspected; in a series of 13 neurosarcoidosis patients without specific suspicion on stroke or vasculitis, MRI with vessel-wall imaging showed vascular involvement in 69% of cases.^36^

## Hypothalamic-pituitary involvement

This rare manifestation of sarcoidosis presents with multiple pituitary hormone abnormalities and opticopathy. Only 13% of patients showed improvement in hormone abnormalities in a series of 50 patients.^37^

### Diagnosis

Diagnosing neurosarcoidosis may be difficult due to its non-specific clinical presentation and results of ancillary test results. A thorough history, physical and neurological examination with low threshold for referral to a neurologist is mandatory. The differential diagnosis is extensive, encompassing a range of demyelinating, inflammatory, infectious, and neoplastic disorders, all of which should be excluded during the diagnostic process.

## Imaging of the CNS

In case of clinical signs consistent with a central nervous system lesion (CNS) or meningitis, cranial or spinal MRI with gadolinium show abnormalities in 79% and 50% of patients respectively. Abnormalities on cranial MRI mostly consist of parenchymal lesions (51%) and contrast enhancement of meninges or cranial nerves (46 and 26%) (Fig. 3).^1^ Spinal MRI with gadolinium mostly shows leptomeningeal and/or pachymeningeal enhancement (61% and 23%), intramedullary enhancing lesions (38%) often involving a long spinal segment with proclivity for the dorsal cord.^1^^,^^28^

## Systemic imaging

**Fig. 4:**
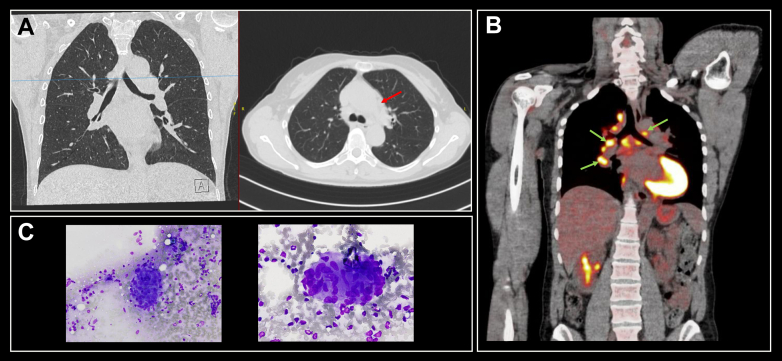
Diagnostics in neurosarcoidosis. A: HR chest CT showing mediastinal lymphadenopathy (pathology: sarcoidosis, red arrow) without pulmonary sarcoidosis in a patient with myelitis. B: 18-FDG PET-CT scan showing multiple enlarged mediastinal and hilar lymphnodes with increased uptake (green arrows). Physiological uptake in the left ventricle. C: fine needle aspirate of lymphnode showing granulomatous inflammation (Giemsa stain).

For detection of pulmonary or lymphoid involvement a chest HR CT scan is the first step, followed by [18F]-fluorodeoxyglucose-positron emission tomography (^18^FDG-PET-CT) in case of a negative result (Fig. 4).^38^ Pulmonary or lymph node sarcoidosis was detected by chest-CT in 70% and by ^18^FDG-PET-CT in 78% in neurosarcoidosis patients.^1^ Although the additional yield of ^18^FDG-PET-CT may appear small, establishing a probable or definite diagnosis can have substantial consequences for clinical decision making and patient discussions. ^18^FDG-PET-CT has superior sensitivity for assessing sarcoidosis activity compared to gallium-67-scintigraphy.^39^

## Cerebrospinal fluid (CSF)

CSF findings are often non-specific with an elevated mononuclear leukocyte count (58%) ranging from 5 to 1571 cells per mm^3^, elevated protein concentration (63%), hypoglycorrhachia (14%), and IgG-index (40%), presence of oligoclonal bands (46%), and elevated ACE (46%).^1^ CSF examination, together with blood serologic tests, are particularly useful to exclude alternative infectious and neoplastic causes.

## Histopathologic confirmation

The diagnosis of sarcoidosis relies on detection of a non-caseating granulomatous inflammatory reaction in the affected tissue. In case of caseating granuloma, (mycobacterial) infection needs to be ruled out. Since biopsy of affected neural tissue can be precarious, a thorough search for other affected organs accessible for biopsy is mandated. In case of mediastinal or hilar lymphadenopathy, endobronchial ultrasound-guided transbronchial needle aspiration is a safe and efficacious procedure to obtain tissue for histologic examination (Fig. 4).^40^ When despite these previous investigations no diagnosis was made, other diagnostic procedures can be considered, such as ophthalmologic and dermatologic assessment, and nerve or muscle biopsy in selected patients. When histopathological evidence of sarcoidosis is found, other diagnoses are not completely excluded, as sarcoid-like reactions can occur in other diseases such as malignancies.

## Diagnostic criteria

In 2018 new diagnostic criteria for neurosarcoidosis were proposed.^41^ In concordance with previous (Zajicek) criteria, patients are classified into three groups: possible, probable, and definite neurosarcoidosis. For all groups, the clinical and diagnostic evaluation should be consistent with neurosarcoidosis. Patients are classified as possible neurosarcoidosis when there is no pathological confirmation, as probable neurosarcoidosis in case of systemic pathologic confirmation, and as definite neurosarcoidosis in case of nervous system pathologic confirmation.^41^ Key principle of the novel criteria is exclusion of infection and malignant neoplasm before diagnosing neurosarcoidosis. The authors of the novel criteria suggest to include only probable and definite cases of neurosarcoidosis for study purposes, to validate these criteria in clinical settings, and update them with new biomarkers.

## Biomarkers

No biomarker has been found yet that is sensitive and specific enough to diagnose neurosarcoidosis.^42^ The best-known biomarkers for sarcoidosis are serum or CSF ACE, lysozyme, and soluble Interleukin 2 receptor (sIL-2r/sCD25). Both ACE and lysozyme come from macrophages or epithelioid cells, whereas sIL-2r is produced by activated T lymphocytes. The value of serum ACE and lysozyme and CSF ACE are limited for diagnosing neurosarcoidosis: sensitivity of serum ACE is 75% in untreated and 35% in treated patients, sensitivity of lysozyme 46% and sensitivity and specificity of CSF ACE is 67% and 67% respectively.^1^^,^^42^

In a diagnostic study on sIL-2r in 11 neurosarcoidosis patients levels in CSF above 150 pg/ml identified untreated NS patients with an overall accuracy of 93%, but only in comparison with non-infectious CNS-diseases and not with infectious CNS disease.^43^ In two of three patients of this similar study with repetitive CSF sIL-2r examinations an increase in sIL-2r preceded the onset of new neurological symptoms.^43^

In a review other biomarkers in neurosarcoidosis such as CSF sIL-2R, IL-6, CD4/CD8 ratio, neopterin, CCL2, S100B, absence of MRZ reaction, KL-6, serum IL-10, Amyloid A1 and plasma S100B are described as candidate biomarkers, but many lack specificity and their value in distinguishing neurosarcoidosis from other infectious or inflammatory diseases is yet unclear.^42^ Possible novel biomarkers for neurosarcoidosis have been found by using proteomics, but these findings need to be confirmed in larger studies.^44^

## Differential diagnosis and neurosarcoidosis mimics

The differential diagnosis of neurosarcoidosis is broad and may contain various infectious, demyelinating, inflammatory, and neoplastic disorders that should be ruled out during the diagnostic process (Table 1). Below we will address some specific mimics of neurosarcoidosis.

**Table 1:** Differential diagnosis in different manifestations of neurosarcoidosis.

Manifestation	Frequency %∗	Diagnoses per causative group
**Meningitis**	12–26	
Leptomeningitis		Infectious: Lyme's disease, Brucella, tuberculosis, fungi (aspergillus)
		Inflammatory: sjögren's syndrome, SLE, rheumatoid meningitis
		Neoplastic: leptomeningeal carcinomatosis
		Other: chemical meningitis (NSAID, IVIG)
Pachymeningitis		ANCA vasculitis, IgG4-related disease, rheumatoid meningitis, meningioma, neurosyphilis, CSF hypotension syndrome.
**Cranial nerve palsies**	37–55	Demyelinating: MS, MOGAD, NMO-SD
		Infections/inflammatory/neoplastic: see section on leptomeningitis
		Other: intracranial hypertension, cerebral venous thrombosis, trauma
**Spinal cord disease**	18–40	Demyelinating disorders: MS, MOGAD, NMO-SD
		Infectious: herpes- (HSV1/2, VZV) entero-, flavi- viruses, HIV, Hep C, HTLV-1, neurosyphilis, Lyme's or Whipple's disease, Brucella, Mycoplasma, Chlamydia, tuberculosis, schistosomiasis
		Inflammatory: Sjögren's syndrome, Behçet's disease, Systemic lupus erythematosus, IgG4-related disease, Langerhans cell histiocytosis
		Neoplastic: primary spinal tumours; meningeoma; solid cancer metastasis/leptomeningeal carcinomatosis; leukemia/lymphoma, paraneoplastic myelitis
		Other: Vitamin B12 deficiency, spinal dural arteriovenous malformation, spondylotic compressive myelopathy, radiation therapy, syringomyelia
**Parenchymal lesion and/or enhancement**	50–67	Demyelinating: MS, MOGAD, ADEM, leukodystrophies (ALD, AMN)
		Infectious: tuberculosis, neurosyphilis, toxoplasmosis, cysticercosis, fungi,
		Inflammatory: Behçet, SLE, sjögren's syndrome, PACNS, CLIPPERS, Susac's syndrome, auto-immune encephalitis
		Neoplastic: primary CNS tumour, meningioma, solid cancer metastasis, leukemia, lymphoma
		Other: granulomatous disease in CVID
**Hypothalamic and/or pituitary involvement**	9–12	Neurosyphilis, tuberculosis, IgG4-related disease, lymphocytic hypophysitis, histiocytosis, pituitary macro-adenoma, germinoma, rathke's cleft cyst, craniopharyngioma, ANCA vasculitis,
**Vascular disease**	6	Atherosclerotic or embolic stroke, neurosyphilis, tuberculosis, VZV, HIV, fungi, systemic vasculitis, PACNS, CAA-ri, APS
**Peripheral nervous system involvement**	5–20	Acute inflammatory neuropathy, chronic axonal idiopathic neuropathy, neuropathy due to diabetes, toxic (alcohol) or metabolic causes
**Myositis**	8–15	Polymyositis secondary to systemic disease, myopathy (genetic, toxic, metabolic causes)

Abbreviations: ADEM, acute disseminated encephalomyelitis; ALD, adrenoleukodystrophy; AMN, adrenomyeloneuropathy; AHL, acute haemorrhagic leucoencephalitis; APS, antiphospholipid syndrome; CAA-ri, cerebral amyloid angipathy related inflammation; CVID, common variable immunodeficiency; CLIPPERS, chronic lymphocytic inflammation with pontine perivascular enhancement responsive to steroids; CVST, cerebral venous sinus thrombosis; NMO-SD, neuromyelitis spectrum disorder; PACNS, primary angiitis of the central nervous system; SLE, systemic lupus erythematosus.

An evident other cause of cerebral parenchymal lesions are malignancies. In literature, many patients have been described with a suspected malignancy such as lymphoma or glioma, who turned out to have neurosarcoidosis and vice versa. In general, lymphoma tends to occur more often in patients with sarcoidosis. This is named the ‘sarcoidosis-lymphoma syndrome’ and thought to be caused by the immunological abnormalities induced by sarcoidosis.^45^ Also, sarcoid-like reactions have been described in the vicinity of tumors.^46^ In addition, different drugs can induce sarcoid-like reactions, examples of these are TNF-α inhibitor, interferon therapeutics, immune checkpoint inhibitors, cancer-targeted therapies, and pulmonary hypertension drugs.^47^

Demyelinating diseases such as multiple sclerosis or MOG antibody associated disease (MOGAD) are another well-known mimic of neurosarcoidosis.^48^ Optic neuritis, cerebral parenchymal lesions, and myelitis in neurosarcoidosis can sometimes be indistinguishable from demyelinating diseases.^49^^,^^50^ CSF markers that help to differentiate are a high CSF protein, pleocytosis, and serum ACE as these are generally normal in MS but do fit with a diagnosis of neurosarcoidosis, whereas intrathecal oligoclonal IgG is more common in MS.^51^

Hypertrophic pachymeningitis (HP) of inflammatory cause may be caused by neurosarcoidosis, but also by granulomatosis with polyangiitis, rheumatoid pachymeningitis, idiopathic HP, and IgG4 related disease.^26^ IgG4-related disease is a fibro-inflammatory disease that can affect any organ but has autoimmune pancreatitis as most common manifestation. Meningeal infiltration (intracranial or intraspinal) is common with a predilection for the dura rather than leptomeninges and sometimes mass lesions. Hypophysitis with secondary hormone deficiencies is also common. Elevated serum IgG4 may point towards the disease, but is normal in 30% of patients.^52^ Histopathologic studies (lymphoplasmacytic infiltrates, storiform fibrosis) and immunostaining (IgG4 positive plasma cells) together with a fitting clinical presentation enable diagnosis of the disease. As in neurosarcoidosis, CNS manifestations may be the sole manifestation of the disease.

Spontaneous intracranial hypotension syndrome (SIHS) can be mistaken for neurosarcoidosis as both can present with diffuse pachymeningeal enhancement on MRI.^53^ Patients with SIHS mostly present with (positional) headache, neck stiffness, and nausea.^54^ Brain sagging is a MRI characteristic that points towards diagnosis of SIHS but also subdural fluid collections and hemosiderosis are characteristic features.^54^ Nodular thickening of meninges does not fit with a diagnosis of intracranial hypotension.

Multiple intracranial meningeomas can be difficult to distinguish from neurosarcoidosis. Gallium-68 dotatate PET-CT scan has been described to improve diagnosis in patients suspect of meningeomas, but increased dotatate uptake has also been described in a neurosarcoidosis patient.^55^^,^^56^ Genetic testing for causes of multiple meningeomas such as neurofibromatosis may aid in establishing the diagnosis.

Pituitary stalk thickening and hypothalamic involvement with diabetes insipidus, meningeal enhancement and skull base lesions are also seen in two other mimics of neurosarcoidosis: Langerhans cell histiocytosis (LCH) and Erdheim Chester Disease (ECD).^57^^,^^58^ These rare histiocytic diseases most commonly present with bone involvement (knee osteosclerosis in ECD, osteolytic skull lesions in 60% of LCH patients) and in contrast to neurosarcoidosis lymph node involvement is rare.^58^ In ECD involvement of the CNS occurs in 40% of patients and mostly manifests as posterior fossa brain parenchyma and spinal cord in addition to aforementioned locations.^58^ Diagnosis is based on histopathology with immunohistochemical staining or detection of BRAF V600E mutation.^58^

Parenchymal granulomatous intracranial disease can occur in patients with common variable immune deficiency (CVID). In a review and cohort study of 19 patients it was shown that CNS lesions in CVID comprise brain mass lesions (70% of patients), leptomeningeal enhancement (10%), white matter lesions (10%) and involvement of the neurohypophysis (10%).^59^ From the 12 patients with histopathology 83% showed granulomatous disease, 50% angiocentric granulomas, vasculitis without granulomas, and lymphocytic infiltrate of the meninges with non-caseating granulomas in 8.3%. Most patients also had lung involvement (73%).

## Treatment

Treatment of sarcoidosis patients is preferably coordinated in a dedicated multidisciplinary team with expertise in treating and monitoring patients on immunomodulatory drugs. For neurosarcoidosis patients, a neurologist with expertise in neurosarcoidosis is preferably involved.^60^ Alertness on involvement of other organ systems is warranted (screening advice is described in sarcoidosis guidelines).^61^

The pharmacological treatment of neurosarcoidosis can be categorized in disease-modifying treatment, with corticosteroid therapy, immunosuppressive medication, and biological therapies, and symptomatic treatment, with anti-epileptic drugs, pain medication, and/or hormonal substitution when indicated. In addition, non-pharmacological interventions might be necessary such as CSF shunting in case of hydrocephalus.

As no randomized clinical trials (RCTs) have been performed on the treatment of neurosarcoidosis, regimens are based on RCTs in sarcoidosis patients or cohort studies and case series of neurosarcoidosis patients (Supplemental Table). In addition, in most studies validated outcome responses are lacking.

**Fig. 5:**
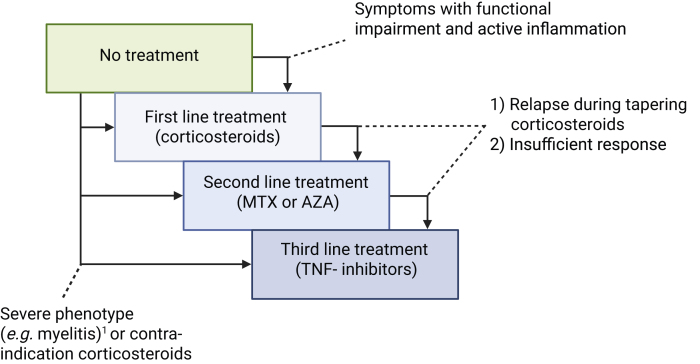
Treatment in neurosarcoidosis. 1. In patients with a severe clinical phenotype, it can be considered to directly start first, second- and third-line treatment. 2. When corticosteroid treatment is contraindicated, or previously significant side-effects have occurred, it can be considered to only use second and third-line treatment. MTX, methotrexate; AZA, azathioprin; TNF-α-inhibitors, tumor-necrosis-factor-alpha inhibitors.

Apart from the 15% of patients with neurosarcoidosis with limited burden of disease or spontaneous recovery, patients will usually start with corticosteroid therapy as first line therapy (Fig. 5).^1^ Favourable outcome on first line corticosteroid therapy was seen in 71% of patients (161/227) with neurosarcoidosis (average follow-up time 4 years).^1^ A cohort of 234 neurosarcoidosis patients showed comparable results with 33% relapse rate overall and neurological relapse rate 23% after monotherapy with corticosteroids.^62^ Corticosteroid treatment regimens consisted of either high-dose oral prednisone (1 mg/kg), tapered after months of treatment, or a combination of three day pulse therapy with 1000 mg/day intravenous methylprednisolone with a lower daily oral prednisone dose (e.g. 20 mg/day).^63^, ^64^, ^65^ Side effects of long-term corticosteroids use are often seen and include osteoporosis, skin atrophy, weight gain, and obesity, diabetes, glaucoma, cataract, avascular bone necrosis, infection, and hypertension.^66^^,^^67^ A meta-analysis of 64 trials showed that pulse corticosteroid therapy for any indication, as compared to a lower dose prednisone (less than 1 mg/kg or 100 mg prednisone or equivalent), no therapy or placebo, was not associated with increased risk for SAEs, the point estimate even favoured pulse therapy. In addition, the occurrence of any adverse event was lower in de pulse group (RR 0.74 (95% CI 0.55–0.99)).^68^

Methotrexate (MTX) and azathioprine (AZA) are often used as second line treatment, other options are mycophenolate mofetil (MMF), (hydroxy) chloroquine (HCL), or cyclosporine A (CSA).^1^ In general, 55% (47/85) of patients have a beneficial response to second line therapy overall.^1^ Evidence lacks on when to start with second line treatments and what treatments to choose. In general, second line therapies are started in corticosteroid refractory disease or as corticosteroid sparing agent in case symptoms recur during or after tapering, but physicians should strongly consider direct initiation in cases with severe CNS involvement as this is associated with higher relapse rates.^69^, ^70^, ^71^

MTX is one of the most commonly used second line therapies.^1^^,^^62^ In cohortstudies clinical improvement with MTX was reported between 19 and 100% of patients and neurological relapse rates between 21 and 47%. Hepatic or hematologic side effects were seen in <2% (10/607) of sarcoidosis patients treated with MTX.^72^ In a recent European guideline on sarcoidosis, MTX is advised as second-line agent of first choice in patients with neurosarcoidosis with continued disease.^73^

AZA is commonly used as second line treatment, but limited evidence exists on its efficacy in neurosarcoidosis (Supplemental Table). Cohortstudies showed clinical improvement with AZA in 22–83% of patients and neurological relapse rates between 22% and 54%. Comparisons between MTX and AZA in different cohort studies are mostly slightly in favour of MTX as second line treatment (100% vs 83% of good response in MTX vs AZA; 54% improvement vs 46%).^51^^,^^74^ Little is known on MMF in neurosarcoidosis, and there is also lack of data for HCQ, CSA and Leflunomide.

Third line treatment consist of TNF-α inhibitor (such as infliximab [IFX] and adalimumab), rituximab, cyclophosphamide or tocilizumab. They have typically been used in severe cases with insufficient response to first-line treatment or in case of deterioration when tapering steroids despite second line treatment.

Since TNF-α is one of the main mediators of sarcoidosis, treatment with a TNF-α inhibitor is currently the most used third line therapy for neurosarcoidosis. Combining results of observational studies with more than five neurosarcoidosis patients shows that IFX led to clinical improvement in 72% (164/227) of patients, stabilization in 21% (40/190), and failure in 7% (17/245) of patients (Table 2). However, studies were not controlled in terms of co-medication and dosage. Switching from original brand IFX or biosimilars did not result in loss of remission or discontinuation.^75^ Important side effect of IFX treatment are infections, these occur in 10–39% of patients and can be serious.^76^

**Table 2:** Treatment response in neurosarcoidosis treated with Infliximab.

Author, year	No of patients	Pathology confirmed (%)	IFX dosage	Median time to IFX initiation (months)	Improvement % (n/N)		Stabilization % (n/N)	Failure % (n/N)	Follow-up time (median, months)
					Complete	Partial			
Gomez et al., 2025	10	NR	5 mg/kg every 4 weeks	NR	100% ‘therapeutic response’			10 (1/10)	12
Dos Santos et al., 2024	27	100	NR	NR	55.5 (15/27)		NR	26 (7/27)	1.19
Morrison et al., 2024	8	100	5 mg/kg every 6–10 weeks	5.7	12.5 (1/8)	75 (6/8)	1/8	0 (0/8)	NR
Hilezian et al., 2021	22	100	5–10 mg/kg/4–10 weeks (most 5 mg/kg/8weeks)	22	60 (13/22)		31 (7/22)	9 (2/22)	24
Fritz et al., 2020	28	100	5 mg/kg every 4 weeks in 18%; 6 weeks 43%, 8 weeks 39%	19	21 (6/28)	50 (14/28)	25 (7/28)	4 (1/28)	NR
Lord et al., 2020	22	100	NR	NR	45 (10/22)		41 (9/22)	14 (3/22)	37
Gelfand et al., 2017	66	100	3–7 mg/kg every 4–8 weeks	2	28.8 (19/66)	48.5 (32/66)	18 (12/66)	3 (2/66)	18
Joubert et al., 2017	28	74.8	5 mg/kg at 0, 2, and 6 weeks and every 8 weeks thereafter	NR	NR	NR	NR	4 (1/28)	96
Cohen et al., 2017	18	100	3–7.5 mg/kg	24	33 (6/18)	56 (10/18)	11 (2/18)	0 (0/18)	6
Riancho-Zarrabeitia et al., 2014	5	80	5 mg/kg iv 0,2,6 weeks and every 6–8 weeks	NR	20 (1/5)	80 (4/5)	0/5	0 (0/5)	NR
Russel et al., 2013	8	100	NR	NR	37.5 (3/8)	25 (2/8)	37.5 (3/8)	0 (0/8)	46.2
Hostettler et al., 2012	6	100	3 mg/kg, in 4-/6-/8-weekly intervals	NR	50 (3/6)	33 (2/6)	0/6	17 (1/6)	36
Moravan et al., 2009	7	100	5 mg/kg on weeks 0, 2, and 6, and then every 6–8 weeks	NR	14 (1/7)	86 (6/7)	0/7	0 (0/7)	24–39
**Total**	**245**	**90 (220/245)**	**NA**	**-**	**Improvement: 72 (164/227)** - complete: 27 (40/146) - partial: 52 (76/146)		**21 (40/190)**	**7 (17/245)**	**NA**

Abbreviations: NA, not applicable; NR, not reported; IFX, infliximab.

In recent years, this stepwise approach is increasingly replaced by experts to a phenotype/severity-centered approach in which TNF-α blockers together with corticosteroids and 2nd line agents are used as first-line agents for specific phenotypes and severe disease (Fig. 5).^60^ Head-to-head comparisons of both treatment approaches are lacking, but arguments for early start of IFX are the substantial relapse rate and morbidity associated with certain phenotypes and the high efficacy of IFX.

Cyclophosphamide is less often used after the introduction of IFX, but might be considered in refractory cases. In a prognostic study 9% (11/120) of patients had a relapse of sarcoidosis while on cyclophosphamide therapy, 8.3% had a neurological relapse.^62^ Other therapies for refractory neurosarcoidosis include adalimumab, rituximab, and cladribine.^77^, ^78^, ^79^, ^80^ In pulmonary sarcoidosis JAK inhibitors have been investigated in a small number of patients, but limited data exists for neurosarcoidosis.^2^

Non-pharmacological treatment of neurosarcoidosis includes CSF shunting in case of hydrocephalus. A review of 36 patients from 33 studies showed 81% required CSF shunting (29 of 36) of which with 72% received a permanent shunt (21 of 36).^27^^,^^81^ In addition, rehabilitation with (when indicated) physio-, occupational and speech therapy are strongly indicated.

## Conclusion

Neurosarcoidosis is a serious form of sarcoidosis that causes substantial morbidity. Diagnosis is often challenging as signs of the disease are non-specific. An accessible site for tissue biopsy has to be searched thoroughly since histopathologic confirmation is essential for future therapies. Corticosteroids remain the cornerstone of immunosuppressive therapy, but additional therapies are often needed.

## Outstanding questions

No consensus exists on the optimal type, timing of initiation, and duration of second or third line therapies, especially in patients with severe disease. Addressing these questions requires RCT's, which necessitate (inter)national collaboration due to the rarity of this disease. Future research should also focus on novel biomarkers to guide selection of patient-targeted therapies, consensus measures for disease activity, functional outcome measurements and their association with novel biomarkers, as well as duration of corticosteroid use and side-effects.

For future patient care and research, centralizing care for neurosarcoidosis patients is critical. This approach will facilitate patients participation in randomised trials aimed at tailoring the optimizing treatment strategies and allow studies investigating the underlying genetic and inflammatory mechanisms. Greater understanding of the disease pathophysiology of neurosarcoidosis is essential for developing novel therapies and improving outcomes for patients.

## Contributors

WF Westendorp was involved in the conception and design of the study, analysis and interpretation of data, and drafting of the manuscript. D. van de Beek and M.C. Brouwer were involved in the conception and design of the study, provided clinical expertise, verification of underlying data (MCB), and critically revised the manuscript for important intellectual content. All authors have read and approved the final version of the manuscript.

## Declaration of interests

The authors report no declarations of interests.
