# Supplementary material for: Neurosarcoidosis: a review from diagnosis and future perspectives
Source: eClinicalMedicine. 2025 Nov 17;90:103653. doi: 10.1016/j.eclinm.2025.103653 (PMC12664804; doi:10.1016/j.eclinm.2025.103653)
Supplement: Supplementary Material [file mmc1.docx]

**Supplementary material**

**Table of contents page**

Supplementary table. Treatment responses in neurosarcoidosis 2-5

References 7-9

| **Supplementary table 1. Treatment responses in neurosarcoidosis** | | | |
| --- | --- | --- | --- |
| **Study** | **No. and type of patients** | **Description of therapy** | **Outcome measure and result** |
| **First line: Corticosteroids (CS)** | | | |
| Fritz *et al*, 2016 (review) | 227 (possible, probable, definite NS) | CS, not further specified | Favorable outcome 71% |
| **Studies on corticosteroids not summarized in review by Fritz et al:** | | | |
| Scott *et al*, 2007^1^ | 19 (definite or probable NS) | CS initiation at 60-80 mg/d, occasionally following high-dose pulse, tapered to 20-30 mg/d by month 4 | Clinical improvement: 35% (7 patients); 55% (11 patients) stable |
| Touati *et al*, 2015^2^ | 18 | CS, not further specified | Improvement: 10 patients.  No improvement: 8 patients |
| Joubert *et al*, 2017^3^ | 234 (definite, probable, possible) | CS, not further specified | Relapse: 33% (85/254 therapeutic sequences); neurological relapse 23% (58/254) |
| Lord et al, 2020 ^4^ | 56 (12 definite, 36 probable, 8 possible) | CS in 51 patients | 37% (19/51) improved, 26% (13/51) stabilized, 37% (19/51) failed |
| Kidd et al, 2020 ^5^ | 166 (biopsy proven) | CS, high dose steroids (40-100mg) | - isolated facial neuropathy: 20/26 recovery (no other IS)  - leptomeningitis: 4/67 improved without relapse on steroid withdrawal, Others additional immune suppression.  For other clinical subgroups no separate effect of CS are given (mostly in combination with other immune suppressive therapies). |
| Bekkour et al, 2023 ^6^ |  | 96.4% CS, CS alone in 54 patients (64%) , CS dose and schedule not further specified | 27.4% full neurological recovery, 28.6% partial improvement, 15.5% stabilization, 28.6% worsened |
| Dos Santos et al, 2024^7^ | 86 (biopsy proven) | 1 mg/kg, 41 pts also CS bolus (250-1000 mg for 3 days) | 16.3% complete and 34.8% partial improvement, 23.3% stabilization, 22.1% relapse, 4 pts progressive worsening despite CS. |
| **Second line: methotrexate, azathioprine, (hydroxyl) chloroquine, mycophenolate mofetil or cyclosporine A** | | | |
| **Methotrexate (MTX)** | | | |
| Soriano *et al*, 1990 ^8^ | 1 probable NS | MTX 25mg/week i.m. | Satisfactory response |
| Lower *et al*, 1997 ^9^ | 28 NS not further specified (no full text) | MTX, not specified | Clinical improvement: 61% (17 patients) |
| Bitoun et al, 2016 ^10^ | 32 definite or probable NS | MTX, median dose 20 mg/wk (range 10-30), combined with CS | Relapse: 47% (NB: comparison with MMF) |
| Joubert *et al*, 2017^3^ | 234 (definite, probable, possible) | MTX 0.3-0.4 mg/kg/d | Relapse: 35% (44/125 therapeutic sequences); neurological relapse 21% (26/125 therapeutic sequences) |
| Arun et al 2020 (suppl data) ^11^ | 80 (‘majority histopatho-logical evidence’) | MTX (with or without CS and/or INF) | 100% (25/25) improvement on mRS |
| Lord et al, 2020^4^ | 56 (12 definite, 36 probable, 8 possible) | MTX, not specified | 19% (5/26) improved, 35% (9/26) stabilized 46% (12/26) failed |
| Sambon et al, 2022 ^12^ | 12 pts | MTX, not specified | Favorable outcome in 67% |
| Kidd et al, 2020^5^ | 56 biopsy proven | MTX, not specified | 134 treated with MTX, outcome overall in this group not specified |
| Dos Santos et al, 2024^7^ | 38 biopsy proven | MTX, not specified, 10 patients also CS | 44.7% improvement or recovery, 15.8% complete recovery. |
| Gomez, 2025^13^ | 24 probable, definite | MTX | 87% therapeutic response, 25% (6/24) relapse |
| **Azathioprine (AZA)** | | | |
| Joubert *et al*, 2017^3^ | 234 (definite, probable, possible) | AZA 50-150 mg/d | Relapse: 57% (8/14); neurological relapse: 43% (6/14) |
| Arun et al, 2020 (suppl data) ^11^ | 80 (‘majority histopatho-logical evidence’) | AZA (with or without CS and/or INF) | 29/35 good response (improvement on mRS), 1/35 no change, 5/35 worsening |
| Lord et al, 2020^4^ | 56 (12 definite, 36 probable, 8 possible) | AZA, not specified | 38% (5/13) improved, 8% (1/13) stabilized 54% (7/15) failed |
| Sambon et al, 2022 ^12^ | 2 pts | AZA, not specified | Favorable outcome in 50% |
| Kidd et al, 2020^5^ | 56 biopsy proven | AZA, not specified | 13 patients, outcome overall in this group not specified |
| Dos Santos et al, 2024 | 9 biopsy proven | AZA, not specified | 0%remission, improvement 22.%, stabilization 44.4%, worsening 11.1%, relapse 22.2%. |
| **(hydroxyl)chloroquine (HCQ)** | | | |
| Joubert *et al*, 2017^3^ | 234 (definite, probable, possible) | HCQ not further specified | Relapse: 27% (16/59 therapeutic sequences); neurological relapse: 20% (12/59) |
| Lord *et al*, 2020 ^4^ | 56 (12 definite, 36 probable, 8 possible) | HCQ not further specified | 1 improved (100%) |
| Sharma *et al,* 1998^14^ | 12 probable or definite NS | Chloroquine phosphate, 250 mg 2 times daily, or hydroxychloroquine sulfate, 200 mg 2 times daily, 6 - 21 months | Stabilization or control of neurologic symptoms in 83% (10/12 patients). No response in 2 patients. |
| **Leflunomide** |  |  |  |
| No studies | | | |
| **Mycophenolate mofetil (MMF)** | | | |
| Joubert *et al*, 2017^3^ | 64 (definite, probable, possible) | MMF 1.0-3.0g/d | Relapse (any): 41% (26/64); neurological relapse: 22% (14/64) |
| Lord *et al*, 2020 ^4^ | 56 (12 definite, 36 probable, 8 possible) | MMF, not specified | 0 improved 25% (2/8) stabilized, 75% (6/8) failed |
| Bitoun *et al*, 2016^10^ | 14 definite or probable NS | MMF median dose 2g/d (range 1-3) | Relapse: 79% |
| Androdias *et al*, 2011 ^15^ | 8 possible, probable NS | MMF, not specified | Remission: 7/8 patients |
| Moravan et al, Neurology 2009 ^16^ | 7 probable or definite refractory NS | MMF 1000 mg PO | Symptomatic improvement in all patients |
| Chaussenot *et al*, 2007 ^17^ | 2 NS not further specified (no full-text) | MMF (2 g/j) | Rapid effective in both cases |
| Dos Santos et al, 2024 | 20 | MMF, not specified | Remission 10.5%, improvement 52.6%, stabilisation 36.8%, worsening 5.3%, relapse 31.6%. . |
| **Cyclosporine A (CSA)** | | | |
| Stern *et al*, 1992 ^18^ | 6 NS, not further specified, refractory | Open lable RCT low vs high dose CSA with CS | Lowering CS dose from 30% to 58%  4 patients deteriorated, 1 died. |
| **Third line: cyclophosphamide and TNF-α blockers (including Infliximab)** | | | |
| **Cyclophosphamide** | | | |
| Lower *et al*, 1997^9^ | 10 NS, not further specified | CPH, not specified | Clinical improvement: 90% (9 patients) |
| Lord *et al*, 2020 ^4^ | 56 (12 definite, 36 probable, 8 possible) | CPH, not specified | 50% improved (1/2) stabilized, 50% failed (1/2) |
| Joubert *et al*, 2017^3^ | 234 (definite, probable, possible) NS | 1g/month | Relapse (any): 9% (11/120); neurologic relapse 8% (10/120) |
| Bigot et al, 2023^19^ | 32, 84% histologically proven | 5-6 month iv. 1000 mg | 48% decrease symptomatic patients (27 to 14), 3 pts complete MRI remission (11.5%), improvement in 8 (30.8%) and stability in 15 (57.7%) |
| Doty *et al*, 2003 ^20^ | 7 definite or probable NS, refractory | CPH mean 5.4 months | 4/7 symptomatic improvement  7/7 objective improvement on MRI/CSF  Mean CS dose reduced from 42 mg/d before therapy to 18 mg/d after therapy. |
| Gomez et al, 2025 | 18 (probable,definite) | CPH monthly infusion 600 to 750 mg/m^2^ | 9/19 (50%) relapse |
| Total |  |  |  |
| **Infliximab** | | | |
| Chaiyanarm et al, 2023^21^ | Systematic review en meta-analysis of 7 studies with 237 patients | 7 studies, 237 patients, 184 received IFX | Proportion of improvement 0.74, relapse rate 0.38. AE in 29.4%, from these 72.2% infections |
| **7 studies included in this systematic review and meta-analysis** | | | |
| Hilezian et al, 2021 | 22 | 5-10mg/kg every 4-10 weeks (most 5mg/kg every 8 weeks | 60% improvement, 31% stabilization, 9% failure |
| Fritz et al, 2020 ^22^ | 28 | 5mg/kg every 4 weeks in 18%, 6 weeks 43% and 8 weeks 39%. | Initial treatment response: complete remission in 21% (6/28), improvement in 50% (14/28), stable disease in 25% (7/28) and 4% (1 patient) deteriorated and died. |
| Lord et al, 2020 ^4^ | 56 (12 definite, 36 probable, 8 possible) | IFX, not specified | 45% (10/22) improved, 41% (9/22) stabilized, 14% (3/22) failed |
| Gelfand *et al*, 2017^23^ | 66 (27 definite, 39 probable) NS | 3-7 mg/kg every 4-8 weeks, median of 1.5 years; combined with CS in 56.1%, MTX in 33.3%, Aza 15.2%, other 3.0%. | Radiologic improvement: 82.1% (of 56 patients with MRI performed): 51.8 complete remission, 30.1% partial improvement  Clinical improvement: 77.3% (complete: 28.8%; partial: 48.5%; stability in 18.2%) |
| Cohen *et al*, 2017 ^24^ | 18 (definite, probable) NS | 3–7.5 mg/kg with CS (n = 18), MTX (n = 15), AZA (n = 2), or MPH (n = 1). | At 6 months: 33% complete remission (6 patients), 56% partial remission (10 patients) 11% (2 patients) stable disease. |
| Riller *et al,* 2019 ^25^ | 20 (definite) NS | With CS (18 patients) MTX and / or AZA (16 patients) | Relapse: 6 patients in 25 months |
| Chakales et al, 2022 | 26 (probable, definite) NS | IFX, not specified | (7/8 patients) 87.5% resulted in remission |
| **Other studies not included in systematic review and meta-analysis** | | | |
| Dos Santos et al, 2024^7^ | 27 biopsy proven | IFX, not specified | 55.5% improvement of symptoms, 1 patient complete recovery. 7/27 new lesions (2 cases myelitis). |
| Morrison et al,2024 ^26^ | 8, 100% | 5mg/kg every 6-10 weeks | 1/8 complete improvement 6/8 partial improvement 1/8 stabilisation |
| Hanset et al, 2021 ^27^ | 1 | IFX, not specified |  |
| Sambon et al, 2022 ^12^ | 2 patients | IFX, not specified | 100% favorable outcome |
| Malik et al, 2020^28^ | 1 | IFX, not specified | improvement |
| Joubert *et al*, 2017^3^ | 28 (definite, probable, possible) NS | 5 mg/kg at 0,2,6 weeks and every 8 weeks thereafter | Relapse (any): 14% (4/28), neurologic relapse: 4% (1/28) |
| Sano et al, 2017 | 1 definite NS | IFX (with CS, MTX, AZA) | Reduction size lesions and improvement of symptoms |
| *Tavee et al, 2017* | *115 small fibre neuropathy* | IFX, not specified | *8/12 symptomatic improvement on IFX 10/14 IVIG and IFX* |
| Riancho-Zarrabeitia *et al,* 2014 ^29^ | 5 possible and probable NS | IFX 5mg/kg 0,2,6 w and every 6-8w, in 3 cases combined with ALM 40mg every other week. | Favorable response: 100% (5 cases) |
| Lorentzen et al, 2014 ^30^ | 1 severe NS | IFX, not specified | Immediate and ramatic response |
| Sohn et al, 2014 ^31^ | 20 IFX treated spinal cord NS biopsy proven | IFX, not specified | No association of final mRS score with IFX therapy (no data reported of patients separately) |
| O’Reilly et al 2013 ^32^ | 1 biopsy proven | IFX, not specified | Complete recovery and radiographic disease remission |
| Russel *et al*, 2013 ^33^ | 8 probable or definite NS | IFX, not further specified | 8 CNS cases from whom 3 had disease activity resolved, 2 had sustained improvement and 3 had unchanged disease activity |
| Hostettler *et al,* 2012 ^34^ | 6 NS, not further specified | IFX 3 mg/kg, in 4-/6-/8-weekly intervals. With CS or other immunosuppressant. | 3 had complete remission, 2 partial remission and 1 no response |
| Croft et al, 2012^35^ | 3 biopsy proven | 3–5 mg/kg body weight administered intravenously at 0, 2 and 6 weeks and then 5 mg/kg every 8 weeks | All 3 clinical and radiographic response |
| Aguiar et al, Rev Port Pneumol 2011 ^36^ | 3 biopsy proven | IFX 5mg/kg at 2,4 and every 8 weeks for 1 year | 1 complete recovery, 1 significant improvement, 1 lack of improvement. |
| Pereira et al, 2011 ^37^ | 3 | 5 mg/kg 2–4 weekly for the ﬁrst8 weeks then maintained on 3–5 mg/kg 8 weekly with follow-up of 6–16 months | Clinical response, limited side-effects |
| Lidar et al, 2010 ^38^ | 2 spinal cord sarcoidosis, biopsy proven | 300 mg every other month or 6 weeks | 1 improved with residual deficit, 1 considerable improvement |
| Santos et al, 2010^39^ | 4 patients, biopsy proven | 3-5 m/kg  body weight, administered intravenously, at 0, 2  and 6 weeks and then every 8 weeks. | All improvement or stabilization of neurological condition |
| Chintamaneni *et al*, 2010 ^40^ | 1 refractory NS not further specified (no fulltext) | IFX, not specified | Successful treatment, relapse after discontinuation |
| Jounieaux et al, Rev Mal Respir 2010^41^ | 8 cases, biopsy proven | IFX 0,2,6 weeks | 50% beneficial response (4/8) (improvement clinical symptoms, MRI or LP) |
| Panselinas et al, 2009 ^42^ | 5 (CNS) | 5 mg/kg iv at 0, 2 and 6 weeks and then every 6 weeks | 1 resolution with normal MRI of brain, 1 improvement symptoms and MRI brain, 2 clinical improvement, 1 stable (visual defects) |
| Moravan et al, 2009 ^16^ | 7 biopsy proven | 5 mg/kg on weeks 0, 2, and 6, and then every 6–8 weeks thereafter | All patients symptomatic improvement and improvement MRI. No SAE |
| Dolhun et al, 2009 ^43^ | 1 probable NS (spinal) | 5 mg/kg every 2 weeks for 3 doses | Clinical improvement, normalization MRI |
| Ritzenthaler et al, 2009 ^44^ | 1 probable NS | IFX 5 mg/kg, week 0, 2, 6 thereafter every 8 weeks | Clinical and radiological response |
| Judson *et al*, 2008 ^45^ | 9 patients with CNS involvement , 11 patients with PNS involvement. Probable or definite NS | RCT placebo or IFX 3mg/kg or IFX 5 mg/kg | CNS: sarcoidosis organ score (SOS) 3 to 5 in week 24 for placebo vs 6 to 5 for Infliximab.  PNS: SOS 4 to 4 for placebo vs 7 to 6 for Infliximab. |
| Sodhi et al, 2008 ^46^ | 4, 3 biopsy proven | 3 mg/kg | Rapid and substantial reversal of clinical course and radiologic findings |
| Saleh et al, 2006^47^ | 4 (2 optic neuritis, 2 CNS) | IFX 3mg/kg 2,4,6,10,14 | All improved |
| Toth et al, 2007^48^ | 1 biopsy proven | mg/kg, with loading doses at weeks 0, 2 and 6, and every 8 weeks thereafter. | Significant improvement with near resolution on MRI |
| Kobylecki et al, 2007 ^49^ | 1 probable NS | 5 mg/kg at 0,2,6 weeks and 6 weeks thereafter | Improvement of symptoms, lowering of CS dose |
| Kumar et al, 2007 ^50^ | 1 definite NS | IFX, not specified | Improvement of symptoms and imaging |
| Salama et al, 2006^51^ | 1 probable NS | IFX, not specified | Disease remission, improvement vision |
| Doty *et al*, 2005 ^52^ | 1 NS not further specified | 5mg/kg at 0,2,8 wk and every 8 weeks thereafter with CS 10 mg/d | Improvement of complaints and MRI abnormalities. |
| Pritchard et al, Ann rheum Dis 2004 ^53^ | 1 biopsy proven | IFX 3/mg/kg 0,2,6 weeks, later 6 mg/kg | Improved, reduction of prednisone |
| Sollberger et al, 2004^54^ | 1 probable NS | 5 mg/kg 2,8,14,20,26 weeks | Clinical improvement and complete resolution of MRI lesions |
| Katz et al, 2003^55^ | 1 biopsy proven | 1 dose 3 mg/kg, 2^nd^ after 10 months with monthly infusions thereafter | Stabilization, vision OD improved en stabilized. |
| Carter et al, 2004 ^56^ | 1 definite NS | 5 mg/kg at 0, 2, and 6weeks and then every 8 weeks | Clinical improvement, MRI dramatic improvement |
| Pettersen et al, 2002 ^57^ | 1 NS | 5 mg/kg and was repeated at 2 and 6 weeks | Clinical and radiologic improvement |
| **Other anti-TNF-α therapies** | | | |
| Dos Santos et al, 2024 ^7^ | 9 pts | Adalimumab (ALM), not specified | Improvement in 4/9 pts, 2/9 complete regression. 2/9 new lesions on MRI. |
| Shen et al, 2024 ^58^ | 10 pts | ALM, 40 mg s.c. every two weeks, without a loading dose | 8/10 positive response clinically and radiographically |
| Hutto et al, 2022 ^59^ | 7 pts + review 4 cases below: 10 pts | ALM | 5/10 improvement, 3/10 remission maintained, 2/10 remained stable without worsening |
| Marnane *et al*, 2009^60^ | 1 probable NS | ALM 40 mg s.c. every other week | Successful treatment, patient asymptomatic |
| Riancho-Zarrabeitia *et al*, 2014 | 3 NS (not further specified, no full-text) | IFX 5mg/kg 0,2,6 w and every 6-8w, in 3 cases combined with ALM 40mg every other week. | Favorable response 100% (3 cases) |
| Metyas *et al*, 2014 ^61^ | 1 probable NS | CS 20 mg, ALM 40mg s.c. every 2 weeks | Marked improvement, discontinuation CS |
| **Infliximab biosimilar** | | | |
| Riller *et al,* 2019 ^25^ | 20 (definite) NS | With CS (18 patients) MTX and / or AZA (16 patients) | Relapse: 6 patients in 25 months |
| **Other (combination) therapies** | | | |
| Scott *et al*, 2007^1^ | 26 definite or probable NS | CS and 1 or multiple of the following:  AZA 150-200 mg/d, 4 weaned during 12-36 months (9 patients) or cyclophosphamide monthly pulses at 600-800 mg/m2 for 3-6 months (6 patients) or MTX 7.5-15 mg/week, 8 patients weaned throughout 12-36 months (18 patients) | Clinical improvement: 69% (18 patients) improved, 15% stable (4 patients), 15% worsening (4 patients) |
| Tikoo et al, 2004 ^62^ | 1 probable NS | Cladribine iv every 3 weeks (20mg daily for 2 days), for 5 cycles. | Dramatic radiological improvement. |
| Lord et al, 2020 ^4^ | 56 patients | Rituximab (RTX) | 0 improved, 50% (2/2) stabilized 50% (2/2) failed |
| Zella et al, 2018 ^63^ | 3 probable NS | RTX 250-500 mg every 6-9 months | Successful treatment in these 3 patients |
| Bomprezzi et al, 2010 ^64^ | 1 definite NS | RTX 1000 mg i.v. 2 doses 2 weeks apart, thereafter every 6 months. | Successful treatment |
| Abbreviations: ALM: Adalimumab; AZA: azathioprin; CS: corticosteroids; CPH: cyclofosphamid; CSA: cyclosporin A; HCQ: (hydroxyl)chloroquine; IFX: Infliximab; i.m.: intramuscularly; i.v.: intravenously; MMF: Mycophenolate mofetil; MTX: methotrexate; NS: neurosarcoidosis; s.c: subcutaneous | | | |

**References**

1. Scott TF, Yandora K, Valeri A, Chieffe C, Schramke C. Aggressive therapy for neurosarcoidosis: long-term follow-up of 48 treated patients. *Archives of neurology* 2007; **64**(5): 691-6.

2. Touati N, Mansour M, Bedoui I, et al. [Neurologic manifestations of sarcoidosis: A study of 18 cases]. *Revue neurologique* 2015; **171**(11): 773-81.

3. Joubert B, Chapelon-Abric C, Biard L, et al. Association of Prognostic Factors and Immunosuppressive Treatment With Long-term Outcomes in Neurosarcoidosis. *JAMA neurology* 2017; **74**(11): 1336-44.

4. Lord J, Paz Soldan MM, Galli J, et al. Neurosarcoidosis: Longitudinal experience in a single-center, academic healthcare system. *Neurol Neuroimmunol Neuroinflamm* 2020; **7**(4).

5. Kidd DP. Sarcoidosis of the central nervous system: Safety and efficacy of treatment, and experience of biological therapies. *Clin Neurol Neurosurg* 2020; **194**: 105811.

6. Bekkour I, Courtin E, Dulau-Metras C, Duffau P, Kremer L, Mathey G. Defining the course of neurosarcoidosis according to presentation at onset and disease modifying treatment: a cohort study of 84 patients. *Therapeutic advances in neurological disorders* 2023; **16**: 17562864231205954.

7. Dos Santos A, Courtin E, Ruet A, et al. Neurosarcoidosis: Clinical, biological, and MRI presentation of central nervous system disease in a national multicenter cohort. *Brain Behav* 2024; **14**(9): e3443.

8. Soriano FG, Caramelli P, Nitrini R, Rocha AS. Neurosarcoidosis: therapeutic success with methotrexate. *Postgraduate medical journal* 1990; **66**(772): 142-3.

9. Lower EE, Broderick JP, Brott TG, Baughman RP. Diagnosis and management of neurological sarcoidosis. *Archives of internal medicine* 1997; **157**(16): 1864-8.

10. Bitoun S, Bouvry D, Borie R, et al. Treatment of neurosarcoidosis: A comparative study of methotrexate and mycophenolate mofetil. *Neurology* 2016; **87**(24): 2517-21.

11. Arun T, Pattison L, Palace J. Distinguishing neurosarcoidosis from multiple sclerosis based on CSF analysis: A retrospective study. *Neurology* 2020; **94**(24): e2545-e54.

12. Sambon P, Sellimi A, Kozyreff A, et al. Epidemiology, clinical presentation, treatment, and outcome of neurosarcoidosis: A mono-centric retrospective study and literature review. *Front Neurol* 2022; **13**: 970168.

13. Gomez H, Noël N, Schmidt J, et al. Comparing the efficacy and safety of cyclophosphamide, infliximab and methotrexate in neurosarcoidosis: a multicenter retrospective study. *Journal of neurology* 2025; **272**(8): 531.

14. Sharma OP. Effectiveness of chloroquine and hydroxychloroquine in treating selected patients with sarcoidosis with neurological involvement. *Archives of neurology* 1998; **55**(9): 1248-54.

15. Androdias G, Maillet D, Marignier R, et al. Mycophenolate mofetil may be effective in CNS sarcoidosis but not in sarcoid myopathy. *Neurology* 2011; **76**(13): 1168-72.

16. Moravan M, Segal BM. Treatment of CNS sarcoidosis with infliximab and mycophenolate mofetil. *Neurology* 2009; **72**(4): 337-40.

17. Chaussenot A, Bourg V, Chanalet S, Fornari JM, Lebrun C. [Neurosarcoidosis treated with mycophenolate mofetil: two cases]. *Revue neurologique* 2007; **163**(4): 471-5.

18. Stern BJ, Schonfeld SA, Sewell C, Krumholz A, Scott P, Belendiuk G. The treatment of neurosarcoidosis with cyclosporine. *Archives of neurology* 1992; **49**(10): 1065-72.

19. Bigot W, Chapelon-Abric C, Mangin O, et al. Cyclophosphamide for the treatment of central neurosarcoidosis: Retrospective study in 32 patients. *Joint Bone Spine* 2023; **90**(3): 105513.

20. Doty JD, Mazur JE, Judson MA. Treatment of corticosteroid-resistant neurosarcoidosis with a short-course cyclophosphamide regimen. *Chest* 2003; **124**(5): 2023-6.

21. Chaiyanarm S, Satiraphan P, Apiraksattaykul N, et al. Infliximab in neurosarcoidosis: a systematic review and meta-analysis. *Annals of clinical and translational neurology* 2023.

22. Fritz D, Timmermans WMC, van Laar JAM, et al. Infliximab treatment in pathology-confirmed neurosarcoidosis. *Neurol Neuroimmunol Neuroinflamm* 2020; **7**(5).

23. Gelfand JM, Bradshaw MJ, Stern BJ, et al. Infliximab for the treatment of CNS sarcoidosis: A multi-institutional series. *Neurology* 2017; **89**(20): 2092-100.

24. Cohen Aubart F, Bouvry D, Galanaud D, et al. Long-term outcomes of refractory neurosarcoidosis treated with infliximab. *Journal of neurology* 2017; **264**(5): 891-7.

25. Riller Q, Cotteret C, Junot H, et al. Infliximab biosimilar for treating neurosarcoidosis: tolerance and efficacy in a retrospective study including switch from the originator and initiation of treatment. *Journal of neurology* 2019; **266**(5): 1073-8.

26. Morrison T, Lakusta-Wong T, Roy-Hewitson C, Gosselin J, Nevares A. Tumor Necrosis Factor Inhibitors as Early Steroid-Sparing Therapy for Neurosarcoidosis: A Case Series. *J Clin Rheumatol* 2024.

27. Hanset N, Tsevi MY, Duprez T, et al. Infliximab for relapsing neurosarcoidosis recurring after kidney transplantation: a case report. *Acta Clin Belg* 2021; **76**(2): 149-51.

28. Al Malik YM. Isolated neurosarcoidosis mimicking multiple sclerosis. *Neurosciences (Riyadh)* 2020; **25**(5): 406-11.

29. Riancho-Zarrabeitia L, Delgado-Alvarado M, Riancho J, et al. Anti-TNF-alpha therapy in the management of severe neurosarcoidosis: a report of five cases from a single centre and literature review. *Clinical and experimental rheumatology* 2014; **32**(2): 275-84.

30. Lorentzen AO, Sveberg L, Midtvedt O, Kerty E, Heuser K. Overnight response to infliximab in neurosarcoidosis: a case report and review of infliximab treatment practice. *Clinical neuropharmacology* 2014; **37**(5): 142-8.

31. Sohn M, Culver DA, Judson MA, Scott TF, Tavee J, Nozaki K. Spinal cord neurosarcoidosis. *Am J Med Sci* 2014; **347**(3): 195-8.

32. O'Reilly MW, Sexton DJ, Dennedy MC, et al. Radiological remission and recovery of thirst appreciation after infliximab therapy in adipsic diabetes insipidus secondary to neurosarcoidosis. *QJM : monthly journal of the Association of Physicians* 2015; **108**(8): 657-9.

33. Russell E, Luk F, Manocha S, Ho T, O'Connor C, Hussain H. Long term follow-up of infliximab efficacy in pulmonary and extra-pulmonary sarcoidosis refractory to conventional therapy. *Seminars in arthritis and rheumatism* 2013; **43**(1): 119-24.

34. Hostettler KE, Studler U, Tamm M, Brutsche MH. Long-term treatment with infliximab in patients with sarcoidosis. *Respiration; international review of thoracic diseases* 2012; **83**(3): 218-24.

35. Croft AP, Situnayake D, Khair O, et al. Refractory multisystem sarcoidosis responding to infliximab therapy. *Clin Rheumatol* 2012; **31**(6): 1013-8.

36. Aguiar M, Marçal N, Mendes AC, Bugalho de Almeida A. Infliximab for treating sarcoidosis patients, Portuguese experience. *Rev Port Pneumol* 2011; **17**(2): 85-93.

37. Pereira J, Anderson NE, McAuley D, Bergin P, Kilfoyle D, Fink J. Medically refractory neurosarcoidosis treated with infliximab. *Intern Med J* 2011; **41**(4): 354-7.

38. Lidar M, Dori A, Levy Y, Lidar Z, Chapman J, Langevitz P. Sarcoidosis presenting as "corset-like" myelopathy: a description of six cases and literature review. *Clinical reviews in allergy & immunology* 2010; **38**(2-3): 270-5.

39. Santos E, Shaunak S, Renowden S, Scolding NJ. Treatment of refractory neurosarcoidosis with Infliximab. *J Neurol Neurosurg Psychiatry* 2010; **81**(3): 241-6.

40. Chintamaneni S, Patel AM, Pegram SB, Patel H, Roppelt H. Dramatic response to infliximab in refractory neurosarcoidosis. *Annals of Indian Academy of Neurology* 2010; **13**(3): 207-10.

41. Jounieaux F, Chapelon C, Valeyre D, et al. [Infliximab treatment for chronic sarcoidosis--a case series]. *Rev Mal Respir* 2010; **27**(7): 685-92.

42. Panselinas E, Rodgers JK, Judson MA. Clinical outcomes in sarcoidosis after cessation of infliximab treatment. *Respirology* 2009; **14**(4): 522-8.

43. Dolhun R, Sriram S. Neurosarcoidosis presenting as longitudinally extensive transverse myelitis. *Journal of clinical neuroscience : official journal of the Neurosurgical Society of Australasia* 2009; **16**(4): 595-7.

44. Ritzenthaler T, Gonzalez-Martinez V, Guegen A, Tilikete C, Vighetto A. [Refractory neurosarcoidosis and infliximab: a growing experience]. *Revue neurologique* 2009; **165**(2): 197-200.

45. Judson MA, Baughman RP, Costabel U, et al. Efficacy of infliximab in extrapulmonary sarcoidosis: results from a randomised trial. *The European respiratory journal* 2008; **31**(6): 1189-96.

46. Sodhi M, Pearson K, White ES, Culver DA. Infliximab therapy rescues cyclophosphamide failure in severe central nervous system sarcoidosis. *Respiratory medicine* 2009; **103**(2): 268-73.

47. Saleh S, Ghodsian S, Yakimova V, Henderson J, Sharma OP. Effectiveness of infliximab in treating selected patients with sarcoidosis. *Respiratory medicine* 2006; **100**(11): 2053-9.

48. Toth C, Martin L, Morrish W, Coutts S, Parney I. Dramatic MRI improvement with refractory neurosarcoidosis treated with infliximab. *Acta neurologica Scandinavica* 2007; **116**(4): 259-62.

49. Kobylecki C, Shaunak S. Refractory neurosarcoidosis responsive to infliximab. *Practical neurology* 2007; **7**(2): 112-5.

50. Kumar G, Kang CA, Giannini C. Neurosarcoidosis presenting as a cerebellar mass. *J Gen Intern Med* 2007; **22**(9): 1373-6.

51. Salama B, Gicquel JJ, Lenoble P, Dighiero PL. Optic neuropathy in refractory neurosarcoidosis treated with TNF-alpha antagonist. *Can J Ophthalmol* 2006; **41**(6): 766-8.

52. Doty JD, Mazur JE, Judson MA. Treatment of sarcoidosis with infliximab. *Chest* 2005; **127**(3): 1064-71.

53. Pritchard C, Nadarajah K. Tumour necrosis factor alpha inhibitor treatment for sarcoidosis refractory to conventional treatments: a report of five patients. *Ann Rheum Dis* 2004; **63**(3): 318-20.

54. Sollberger M, Fluri F, Baumann T, et al. Successful treatment of steroid-refractory neurosarcoidosis with infliximab. *Journal of neurology* 2004; **251**(6): 760-1.

55. Katz JM, Bruno MK, Winterkorn JM, Nealon N. The pathogenesis and treatment of optic disc swelling in neurosarcoidosis: a unique therapeutic response to infliximab. *Archives of neurology* 2003; **60**(3): 426-30.

56. Carter JD, Valeriano J, Vasey FB, Bognar B. Refractory neurosarcoidosis: a dramatic response to infliximab. *Am J Med* 2004; **117**(4): 277-9.

57. Pettersen JA, Zochodne DW, Bell RB, Martin L, Hill MD. Refractory neurosarcoidosis responding to infliximab. *Neurology* 2002; **59**(10): 1660-1.

58. Shen J, Sugita M, Linares-Lopez A, Shah S, Eckstein C, Lackey E. Adalimumab as treatment for neurosarcoidosis: A case series. *Journal of the neurological sciences* 2024; **460**: 123018.

59. Hutto SK, Kyle K, Cavanagh JJ, Reda H, Venna N. Adalimumab for CNS sarcoidosis: single-center experience and literature review. *Journal of neurology* 2022; **269**(4): 2064-72.

60. Marnane M, Lynch T, Scott J, Stack J, Kelly PJ. Steroid-unresponsive neurosarcoidosis successfully treated with adalimumab. *Journal of neurology* 2009; **256**(1): 139-40.

61. Metyas S, Tawadrous M, Yeter KC, Arkfeld DG. Neurosarcoidosis mimicking multiple sclerosis successfully treated with methotrexate and adalimumab. *International journal of rheumatic diseases* 2014; **17**(2): 214-6.

62. Tikoo RK, Kupersmith MJ, Finlay JL. Treatment of refractory neurosarcoidosis with cladribine. *The New England journal of medicine* 2004; **350**(17): 1798-9.

63. Zella S, Kneiphof J, Haghikia A, Gold R, Woitalla D, Thone J. Successful therapy with rituximab in three patients with probable neurosarcoidosis. *Therapeutic advances in neurological disorders* 2018; **11**: 1756286418805732.

64. Bomprezzi R, Pati S, Chansakul C, Vollmer T. A case of neurosarcoidosis successfully treated with rituximab. *Neurology* 2010; **75**(6): 568-70.
